# Role of Circulating Lymphocytes in Patients with Sepsis

**DOI:** 10.1155/2014/671087

**Published:** 2014-08-28

**Authors:** Raul de Pablo, Jorge Monserrat, Alfredo Prieto, Melchor Alvarez-Mon

**Affiliations:** ^1^Intensive Care Unit, University Hospital “Príncipe de Asturias”, University of Alcala, Alcala de Henares, 28805 Madrid, Spain; ^2^Laboratory of Immune System Diseases and Oncology, National Biotechnology Center (CNB-CSIC) Associated Unit, Department of Medicine and Medical Specialties, University of Alcala, 28871 Madrid, Spain; ^3^Immune System Diseases and Oncology Service, University Hospital “Príncipe de Asturias”, University of Alcala, Alcala de Henares, 28805 Madrid, Spain

## Abstract

Sepsis is a systemic inflammatory response syndrome due to infection. The incidence rate is estimated to be up to 19 million cases worldwide per year and the number of cases is rising. Infection triggers a complex and prolonged host response, in which both the innate and adaptive immune response are involved. The disturbance of immune system cells plays a key role in the induction of abnormal levels of immunoregulatory molecules. Furthermore, the involvement of effector immune system cells also impairs the host response to the infective agents and tissue damage. Recently, postmortem studies of patients who died of sepsis have provided important insights into why septic patients die and showed an extensive depletion of CD4 and CD8 lymphocytes and they found that circulating blood cells showed similar findings. Thus, the knowledge of the characterization of circulating lymphocyte abnormalities is relevant for the understanding of the sepsis pathophysiology. In addition, monitoring the immune response in sepsis, including circulating lymphocyte subsets count, appears to be potential biomarker for predicting the clinical outcome of the patient. This paper analyzes the lymphocyte involvement and dysfunction found in patients with sepsis and new opportunities to prevent sepsis and guide therapeutic intervention have been revealed.

## 1. Introduction

Sepsis is a systemic inflammatory response that occurs during infection [1]. Septic shock is the leading cause of multiple organ failure and death in intensive care units, and the incidence is increasing worldwide [2–4]. The pathogenesis of sepsis is a result of a complex network of events involving immune-inflammatory and anti-inflammatory processes triggered by the infection agent [5]. This host response is complex and variable, in which both proinflammatory and anti-inflammatory mechanisms can contribute to either clearance of infection and tissue recovery or organ injury. Early and appropriate intervention is critical for improving the patient's outcome, reducing morbidity and mortality [6]. It is generally accepted that the clinical strategy for improving the outcome of sepsis patients includes the advancement in the knowledge of the pathogenesis of this syndrome as well as the identification of biomarkers to establish risk assessment, predicting the development of individual or multiple organ dysfunctions, guiding antimicrobial therapy, and establishing new and individualized treatments.

Sepsis is initiated when the host responds to pathogen insult. The first line of defenses is constituted by the innate immune system response. Several effector cells are involved in this antimicrobial response including different leukocyte populations. Monocyte-macrophage cells and dendritic cells play a key role in the innate immune response. These cells have the ability to phagocytose bacteria and interact with their products through an interaction with their pattern-recognition receptors. These activated phagocytic cells release proinflammatory mediators, such as cytokines, chemokines, lipid mediators, nitric oxide, and oxygen radicals [7–9]. Activated neutrophils also promote clearance of bacteria, and they subsequently contribute to tissue inflammation and injury through respiratory burst, cytotoxicity, degranulation, increased vascular permeability, and organ injury by releasing several proinflammatory mediators, myeloperoxidases, and proteases [10]. Thus, a “cytokine storm” is generated, which is responsible for triggering the inflammation. But the immune system, including cells of the adaptive immune response, may also harbor humoral and cellular mechanisms that attenuate the potentially harmful effects of the proinflammatory response. However, the release of anti-inflammatory cytokines also appears to be exacerbated, as illustrated by the strong relationship between high levels of these mediators and poor outcome [11]. Indeed, our group and other researchers have described that an early response to continuously elevated anti-inflammatory cytokine serum levels was better predictor of mortality than the classic proinflammatory cytokines in patients with septic shock [12]. It is important to remark that this compensatory anti-inflammatory response syndrome named CARS is a two-wave process that follows SIRS (systemic inflammatory response syndrome) in experimental animals, but in most of patients both events are concomitant, [13] and it is often found when patients are admitted to the ICU [12]. Furthermore, the adaptive immune response has a relevant role to control of bacterial infection [14]. Adaptive immunity is driven by innate immune cells through sensing microorganisms and presenting antigens in the context of major histocompatibility complex class II (MHC class II) and costimulatory molecules. The recent discovery of subsets of lymphocytes that are defined by their limited antigen receptor variability and are restricted to specific tissue may prove a link between immune activation and antibacterial defense during sepsis [15]. “Innate lymphocytes” are defined by their limited antigen receptor variability, and, therefore, these T cells have a memory phenotype in the absence of deliberate immunization [16]. The innate-like lymphocytes include natural killer T cells, gamma delta T cells, and mucosal-associated invariant T (MAIT) cells. MAIT cells are already primed to gastrointestinal flora and work in cooperation with the innate response to stave off infections [15]. Furthermore, T lymphocytes play a critical role in the regulation of antimicrobial phagocytic and cytotoxic activity of the innate immune response cells [17]. Interferon (IFN)-*γ* and granulocyte macrophage colony stimulating factor (GM-CSF), mainly produced by T lymphocytes, increase this defensive activity but other cytokines such as interleukin (IL)-10 have inhibitory effects [17, 18]. B lymphocyte response also plays an important role in the defensive host response. B cells produce cytokines, present antigens to T lymphocyte, and differentiate into antibody producing cells [19]. Antibodies bounded to bacteria may increase bacteria opsonization and favor phagocytosis [17]. However, abnormal bacterial induced activation of T and B cells may be followed by inflammation and endothelial and tissue damage [20, 21].

Blood lymphocyte dysfunction during sepsis has long been recognized with significant lymphopenia and decreased lymphocyte T CD4+, CD8+, and natural killer (NK) cells [5]. However, recently a renewed interest in lymphocyte dysfunction during sepsis emerged from studies demonstrating that immunosuppression was present not only in peripheral blood cells but also locally in organs in patients who died of sepsis [22].

In this review, we try to highlight the role of the main populations of blood lymphocytes in sepsis and we discuss how different kinetic patterns of lymphocyte subsets are involved and their relationship to the surviving outcome. This knowledge in the future may have important therapeutic implications for patients with septic shock. Furthermore, circulating lymphocyte abnormalities might have also potential prognostic biomarker signification.

## 2. Lymphopenia and Anergy

B- and T-lymphopenia is a hallmark of sepsis that can be mimicked in human volunteers receiving a bolus of lipopolysaccharide [11]. Extensive lymphocyte apoptosis is seen in animal models of sepsis and in patients with sepsis [23]. In a model of cecal ligation and puncture, prevention of lymphocyte apoptosis with caspase inhibitors results in a marked improvement in animal surviving [24]. Other studies on animals also suggest that immune depression resulting from the loss of lymphocytes may be the key factor in inability to survive sepsis [24–27].

Anergy is a tolerance mechanism in which the lymphocyte is intrinsically functionally inactivated following an antigen encounter, but it remains alive for an extended period of time in a hyporesponsive state [5]. T-cell anergy relates to a decreased proliferation to mitogen stimulation, a shift toward a T_H2_ profile of cytokine secretion, an increased apoptosis, and an increased percentage of CD4+CD25+ regulatory T lymphocytes (Tregs) [28]. The occurrence of a state of lymphocyte anergy has been described in patients with major trauma or burns, associated with mortality rate and with the development of secondary septic complications [5, 29]. Meakins et al. described that surgical patients who had failure of delayed hypersensitivity response had an increased risk of sepsis and related mortality [30]. Therefore, defective T-cell proliferation and secretion of IL-2 and TNF correlated with sepsis mortality [31].

## 3. T Lymphocytes

T-cell compartment plays a critical role in regulating the effector stage of the immune response. CD3+CD4+ T lymphocytes or T helper (T_H_) cells are mainly involved in the regulation of the immune response [32]. 

It has been recognized that different CD4+ T cells subsets play a critical role in response to microbial challenges. The first subsets recognized were denoted by T_H1_ and T_H2_ cells based on the selective production of 2 cytokines, IFN-*γ* and IL-4, respectively [33]. T_H1_ cytokines exert a positive feedback on antigen-presenting cells, whereas T_H2_ cytokines promote downregulation of the immune response. This T_H1_ paradigm was reasonably useful for the initial categorization of mechanisms involving elimination of microbial pathogens. Previous works showed a shift from T_H1_ to T_H2_ cytokine profiles following severe aggression such as trauma, burns, cardiac arrest, and infection [14, 34].

It has been proposed that the lack of a shift from T_H1_ to T_H2_ response increases survival among patients with sepsis [35]. Reductions in circulating CD4+ T-lymphocytes and their shift to a T_H2_ phenotype characterize aspects of sepsis-induced immunosuppression [36]. The associations between complicated clinical course and unfavorable prognosis of septic patients with the decline of peripheral blood CD4+ T-lymphocytes were established in a majority of trauma victims or surgical patients with secondary sepsis [37, 38]. We have described that T lymphopenia found in patients with septic shock persisted during the first week of follow-up in the intensive care unit (ICU) and was independent of the outcome [39]. These findings are consistent with reports demonstrating significant lymphopenia early in the course of disease [40, 41]. However, at the end of the second week of follow-up, we observed that the absolute number of circulating CD3+CD4+ T cells had clearly normalized in surviving patients with septic shock [36].

Nowadays, we know that the opportunities for T_H_ lymphocytes diversity are far greater than just T_H1_ and T_H2_ profiles. The newly described T_H_ cell subsets include T_H17_, T_H9_, and T_H22_ cells; follicular helper T (T_FH_) cells; and different types of regulatory T (Treg) cells [42].

T_H17_ has relatively recently been characterized as an IL-17-producing subset of CD4+ T cells. Naive CD4+ T cells, in the presence of IL-6, IL-21, or TGF-beta, can proceed to a T_H17_ phenotype [43, 44]. Their proliferation and differentiation are supported by IL-23 and IL-1 secreted from antigen-presenting cells [43, 45]. Once differentiated, T_H17_ cells are capable of producing not only IL-17, but also IL-21, IL-22, TNF-α, and IFN-*γ* [46–48], suggesting plasticity of these cells, with an ability to produce different cytokines depending upon environmental stimulus [46, 48]. IL-17 plays a major role in linking adaptive and innate immune responses. IL-17 is a potent proinflammatory cytokine which induces the production of many other proinflammatory cytokines, chemokines, and other inflammation mediators such as prostaglandin E2 and nitric oxide [49]. Information about its role in human sepsis is scarce [14]. T_H17_ cells contribute to host defense against extracellular bacteria, such as* Staphylococcus aureus* and* Klebsiella pneumoniae* as well as fungi [50, 51]. In humans, T_H17_ lymphocyte count on day 1 and after 6 days in survivors with severe sepsis was higher than that in nonsurvivors [52]. Salomao et al. observed an increased proportion of CD4+ lymphocytes producing IL-17 in patients with sepsis [14]. Thus, T_H17_ differentiation appears to contribute significantly to the surviving in patients with severe sepsis, and it represents one exception in the overall downregulation of T-cell immune functions in these patients.

Treg is one of the T-cell subsets that have strong immunosuppressive activity, playing an essential role in controlling both adaptive and innate immune responses. These cells can downregulate effector activities mediated by CD4+ T cells, CD8+ T cells, NK cells, and also dendritic cells and B cells [37, 52–57]. Recently, Wu et al. found that the circulatory Treg lymphocyte counts on day 1 were higher in surviving patients with severe sepsis than those in nonsurviving ones [35]. This finding confirms results found by other authors [58, 59]. The relative increase in circulating Treg might play a role in lymphocyte anergy described after septic shock [60]. This, altogether, strongly suggests that Treg cells not only represent a reliable marker of immunoparalysis in sepsis, but also may play an important role in its pathogenesis.

CD8+ T lymphocytes are effector cytotoxic cells. We have found a decrease of CD8+ T lymphocytes in patients with septic shock at ICU admission [36]. In survivors, CD8+ T lymphocytes showed a further drop on day 3 of followup, followed by a gradual recovery although numbers failed to reach the count recorded in healthy controls. Importantly, CD3+CD8+ T lymphocyte count in survivors was significantly diminished with respect to nonsurvivors on day 3. A drop in circulating CD3+CD8+ T cells has been described by other authors [52, 54, 61–63].

CD45 is essentialin T-cell differentiation and antigen receptor signaling [64]. When inflammatory agents activate noneffector CD45RA+CD45RO− T lymphocytes, such as bacterial infection, the isoform CD45RO is upregulated and CD45RA is downregulated [65]. CD28 is a costimulatory molecule that plays a key role in regulating the activation and surviving of T lymphocytes [66–68]. It has been reported that patients with severe sepsis showed a significant reduction in T lymphocyte CD28 expression [69]. The migration of circulating T lymphocytes to peripheral lymph nodes depends on the expression of the CD62L homing receptor [70]. We observed downregulation of L-Selectin expression on CD3+CD8+ cells in patients with septic shock and it was associated with a better prognosis. When we analyzed the phenotype of the circulating CD3+CD8+ T cells according to the activation criteria in patients with septic shock at ICU admission, all patients showed low count of CD3+CD8+CD45RA+CD45RO− T lymphocytes (naïve cells), and survivors also have low CD3+CD8+CD45RA−CD45RO+ lymphocytes (memory cells) at day 3 of the follow-up, associated with a lower count of CD3+CD8+CD28+ T lymphocytes. Furthermore, survivors also show lower count of CD3+CD8+CD62L+ T lymphocytes. These findings may suggest that the rapid migration of activated CD8+ T cells to peripheral lymph nodes may be a mechanism contributing to patient survival and, therefore, delayed tissue response could determine the failure of the immune system in patients with the worst outcome [36]. It is known that cellular immune responses play a critical role in the defense against infections and strong T-cell responses have been reported in patients who clear infection [71].

As we have described above, the innate-like lymphocytes include natural killer T (NKT) cells, gamma delta T (*γ* *δ*-T) cells, and MAIT cells. The main characteristic of these cells is their limited antigen receptor variability, and, therefore, these T cells have a memory phenotype in the absence of deliberate immunization [16]. NKT cells are activationally restricted by the MHC class I-like molecule called CD1d [72]. NKT cells are potent producers of proinflammatory mediators such as IFN-*γ*; they are capable of activating macrophages, NK cells, dendritic cells, and effector T cells and possess cytotoxic effector activity [73]. Altogether, they have been thought to be significant promoters of the dysregulated septic response [74]. Recently, Heffernan et al. have demonstrated that invariant NKT (iNKT) cells, a type of NKT cells that express an invariant Vα24/Jα18 chain and a restricted *β* chain [72], are increased in sepsis and this is most pronounced in geriatric nonsurviving patients [75]. However, Grimaldi et al. did not observe any quantitative changes in circulating NKT cells in critically ill patients with severe infections [76].

*γ* *δ*-T cells are preferentially localized in mucosal organs containing epithelia and are known to regulate macrophages [77]. Circulating *γ* *δ*-T cells count is reduced in patients with sepsis [78, 79], and this reduction seems to become more intense as the septic process becomes more severe [80]. Thus, these studies suggest the key role of *γ* *δ*-T cells in the defense against infection and open up the possibility of initial explorations of new therapeutic strategies [80].

MAIT cells have the ability to be activated in the presence of antigen-presenting cells infected with Gram-positive (except streptococcal and enterococcal bacteria), Gram-negative bacteria and yeasts [81]. They display fast activation upon microbial infection and rapidly express effector mechanisms including high amounts of proinflammatory cytokines production such as INF-*γ* and IL-17. A recent study by Grimaldi et al. showed an early and marked decrease in MAIT cell counts in patients with severe sepsis and a relationship between this reduction throughout the first 4 days of ICU admission and the development of ICU-acquired infections [76]. These findings suggest that MAIT cells are involved in sepsis-induced immunosuppression. Therefore, the understanding of innate-like lymphocytes may be crucial for the development of potential therapies to restore immune system function in patients with sepsis.

## 4. Natural Killer (NK) Cells

Recently, several works have highlighted a key role of natural killer cells during sepsis [82, 83]. NK cells have effector cytotoxic activities and immunoregulatory functions such as the production of cytokines such as IFN-*γ*, TNF-α, and granulocyte-macrophage colony-stimulating factor (GM-CSF) [84, 85]. NK cells are also engaged in crosstalks with other immune cells, such as dendritic cells [86], monocytes, macrophages [87], and neutrophils [88]. Furthermore, NK cells are probably directly involved in the antibacterial response of the innate immune system due to their capacity to recognize pathogen-associated molecular patterns [89]. It is possible that all NK cell subsets are not equivalent in their antibacterial activity [90].

In sepsis, severe lymphopenia also affects circulating NK cells [38, 82, 83]. Andaluz-Ojeda et al. have reported that patients with the highest NK cell number had the lowest probability to survive [83]. However, we do not find higher percentages of NK cells in nonsurviving patients with septic shock in agreement with other authors [82, 91, 92]. Sepsis is also associated with an activation of NK cells. CD69 is rapidly induced in NK cells and its role in NK cytotoxic has been demonstrated in humans [93]. An increase in the counts and percentage of the CD3−CD56+CD69+ cells in nonsurvivors at ICU admission and 48 hours later has been shown [82]. We also found a higher percentage of the expression of CD57, a marker of long-lived and highly differentiated effector NK cells, in patients with septic shock who died. Our data demonstrate that surviving patients with septic shock exhibited more NK cells depletion than nonsurviving ones and that these NK cells are early activated and rapidly differentiated in patients with septic shock.

Functions of circulating NK cells in critically ill patients have been poorly studied. Unexpectedly, and in apparent contradiction with murine data [94–100], Forel et al. found that patients with sepsis exhibited decreased production of IFN-*γ*, especially those who presented with septic shock [92].

## 5. B Cells

Classically, B lymphocytes are characterized by their ability to differentiate into immunoglobulin secreting plasma cells. However, B cells also play critical immunoregulatory roles as antigen presenting cells and also as cytokine producing cells. Currently, it is accepted that B cells have an important role in both adaptive and innate immune responses [101]. During the immune response against infectious agents, the production of antibodies by antigen activated B lymphocyte clones is critical for the efficient eradication of many agents. B cells may also act as effective antigen presenting cells of the microorganism antigens to T lymphocytes [102]. Furthermore, the interaction of several bacterial products with B cells may also cause their activation and cytokine secretory function [101]. Interestingly, the activation of B cells by microorganisms takes place not only by antigen recognition, but also through the activation of Toll-like receptors (TLR). Dual antigen-specific B-cell receptors (BCR) and TLR engagement can fine-tune functional B-cell responses, directly linking cell-intrinsic innate and adaptive immune responses [103]. Moreover, it has been demonstrated that B cells also release a broad variety of cytokines. Pivotal to B cells is IL-10 production, which inhibits proinflammatory cytokines and restrains the excessive inflammatory responses that occur during autoimmune diseases or that can be caused by unresolved infections [104, 105].

The role of B lymphocytes in the pathogenesis of sepsis has not been established. Recently, it has been proposed that B cells are involved in the early innate immune response during experimental bacterial sepsis [101] and Darton et al. reported that adults who have recovered from an episode of invasive pneumococcal disease demonstrate defective B-cell activation [106]. More recently, Rauch et al. have demonstrated that innate response activator B cells (IRA-B cells) play a critical role in the response to sepsis, as mice lacking B-cell-derived GM-CSF are unable to clear bacteria, elicit exaggerated inflammatory responses, and, more likely, succumb to infection [107]. Moreover, they have already developed an in vitro system to expand IRA-B cells from their precursors and then inject them into the patient to boost their immune response [108]. Thus, B cells appear to play a role in the sepsis immunodisturbance [109] and one may expect that, by restoring their function, the overall immune response could be improved.

We have reported that patients with septic shock suffer from a severe retraction of peripheral blood B lymphocytes [110]. Furthermore, circulating B cells show increased expression of CD95 antigen. As described in T cells [111], the increased expression of CD95 on B cells from patients with septic shock might contribute to the observed reduction of circulating B cells in these patients. Several studies have shown an inverse correlation between lymphocyte count and survival [23, 111]. We also found higher percentage of CD95 expression on B cells from nonsurvivors than that from survivors [110].

The retraction of circulating B cells affects heterogeneously the different B-cell subsets in patients with septic shock [100]. In patients with septic shock, the numbers of circulating CD19+CD69+ remain normal, but CD19+CD23+ B lymphocytes are clearly decreased. Furthermore, higher percentages of circulating CD19+CD23+ are associated with better clinical outcome of the patients [110]. CD23 is involved in different regulatory functions such as enhancing antigen presentation, improving B-cell differentiation, and growth [112]. Some authors have reported that CD23 is expressed on activated B cells whereas others have suggested that peripheral blood CD23 B cells resemble classic memory cells [112].

CD80 and CD86 are critical molecules in the B-cell antigen presentation function. In murine studies of sepsis, an important role for CD80 and CD86 antigens in the response to sepsis has been established [113, 114]. Our results showed higher percentage of CD86 expression on B cells from patients with septic shock. Furthermore, at ICU admission, nonsurvivors had more elevated percentages of CD19+CD80+ B cells than those found in survivors [110].

## 6. Peripheral Blood Lymphocytes as Biomarkers in Sepsis

Leukocyte phenotyping might also have a predictive value for the development of immune-supportive or immunostimulatory therapies in the management of septic shock patients [115, 116]. We have also studied the predicting value for the outcome of combining different T- cell, B-cell, and NK cell markers in patients with septic shock. According to cytomics methodology [117], we have found a set of five immunophenotypic variables (CD3+CD8+CD28+, CD3+CD8+CD45RA+CD45RO–, CD19+CD80+, CD56+CD69+, and CD3+CD11A br+CD11B+ lymphocyte subsets) which are able to improve the prediction for outcome in septic shock patients to a sensitivity of 94% and a specificity of 100% [118].

## 7. Therapeutic Approaches for Restoring Lymphocyte Functions

Despite the extraordinary developments in understanding the immunopathology of sepsis, advances in immunotherapy have been very disappointing. Recognizing the pivotal role of lymphocytes in orchestrating the body's immune response against infection, based on their ability to interact with cells of innate and adaptive immune system, it might be possible to decrease mortality in sepsis by therapies to augment host immune response through restoring lymphocytes function.

**Figure 1:**
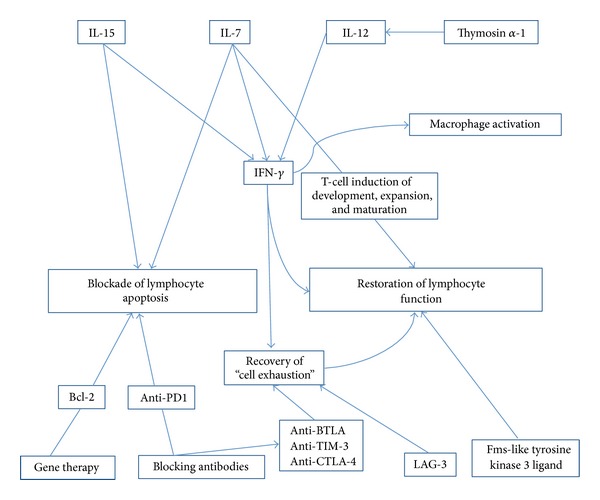
Therapeutics approaches to counteract apoptosis and recovery lymphocyte functions. Based on lymphocyte alterations described in this review, two main therapeutic strategies must be taken into account in patients with sepsis: to block lymphocyte apoptosis for recovery of lymphocyte count or to restore effector lymphocyte functions. Abbreviations: IL: interleukin; IFN: interferon; Bcl-2: B-cell lymphoma 2 gen; PD-1: programmed cell death protein 1; BTLA: B- and T-lymphocyte attenuator; TIM-3: T-cell immunoglobulin and mucin protein 3; CTLA-4: cytotoxic T-lymphocyte-associated protein 4; LAG-3: lymphocyte activation gene 3.

Therapeutic strategies for treating lymphocyte alterations in patients with sepsis should include the restoration of lymphocyte count and function or the blockade of inhibitory signals (Figure 1). Currently, the most promising immunotherapeutic agent is purified interleukin (IL)-7. IL-7 is an essential cytokine that affects both T and B cells and induce T lymphocyte development, survival, expansion, and maturation in humans [119]. In experimental models of sepsis, IL-7 treatment increased the production of CD4 and CD8 cells, restored delayed type hypersensitivity responses, blocked lymphocyte apoptosis, reversed the impaired IFN-*γ* production leading to macrophage activation, increased expression of cell-adhesion molecules leading to improved T cells recruitment to sites of infection, and increased T-cell receptor diversity leading to more potent immunity against pathogens [120]. Furthermore, IL-7 can mediate the crosstalk between T_H1_ and T_H17_ lymphocytes during sepsis such that neutrophil recruitment and bacterial clearance are improved [121].

IL-15 is also a pleiotropic cytokine having promising results in experimental models of sepsis. The administration of IL-15 improved survival in two different models of sepsis and was associated with an increase in lymphocyte survival, decreased apoptosis of NK cells, dendritic cells, and T cells, and increased IFN-*γ* secretion [122].

Fms-like tyrosine kinase 3 ligand is a cytokine capable of enhancing the sensitivity of antigen-specific B and T-cell responses upon bacterial challenge, and it would be another potential treatment in infectious disease [123].

The development of lymphocyte apoptosis is markedly more increased in septic patients than in critically ill nonseptic controls [23]. Strategies to block programmed cell death in lymphocytes are suggested to be beneficial in sepsis [119]. Also, amplified antiapoptotic signals might be of therapeutic value. A genetically manipulated T-cell resistant to apoptosis and polyclonal for a variety of pathogens could be transfused during immune dysfunction to restore patient immunity. For instance, mice transfected with the human gene* Bcl-2* were protected from death after cecal ligation and puncture [24]. Moreover, the transfer of T cells from* Bcl-2* overexpressing mice into wild type septic mice also improved survival [124]. More recently, cell penetrating peptides (CPPs) have been used to deliver the antiapoptotic Bcl-xL-derived BH4 peptide to prevent injury-induced apoptosis both in vitro and in vivo [125]. Furthermore, administration of ritonavir, a HIV protease inhibitor which is known to prevent apoptosis in vitro, improved survival in mice with sepsis [126].

The development of blocking antibodies to multiple inhibitory receptors involved in sepsis represents another innovative therapeutic strategy. PD-1 (programmed death 1) is a negative costimulatory molecule expressed on immune effector cells. It is upregulated in sepsis and impairs immunity by inducing apoptosis, increasing production of IL-10, preventing T-cell proliferation, and causing T-cell exhaustion. Guignant et al. showed that PD-1 overexpression on circulating T cells from patients with sepsis, correlated with decreased T-cell proliferation, increased secondary nosocomial infections and mortality [127]. In animal models of bacterial and fungal sepsis, blockade of the PD-1 pathway improves survival [128–130]. Additional receptors associated with cell exhaustion such as BTLA, TIM-3, LAG-3, and CTLA-4 may be good potential therapeutic targets [131].

IFN-*γ* plays a pivotal role in regulating the adaptive immune response mediated by T-lymphocytes and dendritic cells and in controlling the NK and phagocytic cells of the innate host immune antimicroorganism defense system. Our group showed that surviving patients exhibited significantly higher levels of IFN-*γ* than the healthy controls during the first 14 days of monitoring [12]. Thus, it could be that the increased circulating IFN-*γ* levels noted here in survivors might be linked to a better immune response against the microorganisms causing septic shock. Döcke et al. treated patients with sepsis showing low monocyte HLA-DR expression with IFN-*γ* and observed the recovery of the deficient HLA-DR expression. Clearance of sepsis was achieved in eight out of nine patients. IFN-*γ* is a critical immunoregulatory cytokine [132].

IL-12 is a cytokine that induces expression of the T_H1_ lymphocyte phenotype [133]. IL-12 increased survival in an animal model of burn injury after a septic challenge. It acts, at least in part, through IFN-gamma [134]. On the other hand, thymosin alpha-1 (Tα1) is a molecule with known immunostimulating properties [135], and it can induce T-cell and dendritic cell maduration as well as increasing IL-12 expression. Wu et al. showed a reduction in 28-day mortalities in patients with severe sepsis with an associated increase in mHLA-DR [136].

In this review, we have described the main changes in circulating lymphocytes from patients with sepsis (Table 1). Do not forget that these cells perform their function in peripheral tissues. Boomer studied postmortem spleen and lung tissue from patients with sepsis who died in ICU and they found that circulating blood cells showed similar findings to those in previous studies [22]. However, the analysis of circulating lymphocytes to be clinically relevant has to be confirmed in a large-size population, with standardized methods, [133] and finally—and most importantly—any targeted therapeutic intervention to restore immune system function must be demonstrated in large randomized studies.

## Figures and Tables

**Table 1:** Main lymphocyte types' alterations in sepsis.

Lymphocyte type	Roles	Outcomes in sepsis	References
CD4+ T cells	T_H1_: positive feedback on antigen presenting cells. T_H2_: promoting downregulation.	Lack of a shift from T_H1_ to T_H2_ increases survival.	[35]
	T_H17_: producing IL-17, IL-21, TNF-*α*, and IFN-*γ*.	T_H17_ lymphocyte count in survivors was higher.	[52]
	Treg: immunosuppressive activity.	Treg lymphocyte counts higher in surviving patients	[41]

CD8+ T cells	Effector cytotoxic cells.	In survivors, CD8+ T lymphocytes showed a further drop on day 3 of follow-up.	[36]

NK cells	Effector cytotoxic activity and immunoregulatory function.	CD69+, an activation marker, increases in nonsurvivors. CD57+, a marker of long-lived and differentiated NK cells, shows higher percentage in nonsurvivors.	[72]

B cells	Ability to differentiate into immunoglobulin secreting plasma cells.	An inverse correlation between count and survival, but this relation affects heterogeneously the subsets.	[23, 101]

Abbreviations: T_H_: T helper; IL: interleukin; TNF: tumor necrosis factor; IFN: interferon.
